# Immunotherapy at any line of treatment improves survival in patients with advanced metastatic non‐small cell lung cancer (NSCLC) compared with chemotherapy (Quijote‐CLICaP)

**DOI:** 10.1111/1759-7714.13272

**Published:** 2019-12-12

**Authors:** Alejandro Ruiz‐Patiño, Oscar Arrieta, Andrés F. Cardona, Claudio Martín, Luis E. Raez, Zyanya L. Zatarain‐Barrón, Feliciano Barrón, Luisa Ricaurte, María A. Bravo‐Garzón, Luis Mas, Luis Corrales, Leonardo Rojas, Lorena Lupinacci, Florencia Perazzo, Carlos Bas, Omar Carranza, Carmen Puparelli, Manglio Rizzo, Rossana Ruiz, Christian Rolfo, Pilar Archila, July Rodríguez, Carolina Sotelo, Carlos Vargas, Hernán Carranza, Jorge Otero, Luis E. Pino, Carlos Ortíz, Paola Laguado, Rafael Rosell, on behalf of the CLICaP

**Affiliations:** ^1^ Foundation for Clinical and Applied Cancer Research (FICMAC) Bogotá Colombia; ^2^ Molecular Oncology and Biology Systems Research Group (FOX‐G), Universidad el Bosque Bogotá Colombia; ^3^ Thoracic Oncology Unit, Instituto Nacional de Cancerología (INCan) México City Mexico; ^4^ Clinical and Translational Oncology Group Institute of Oncology, Clínica del Country Bogotá Colombia; ^5^ Medical Oncology Department, Thoracic Oncology Section Instituto Fleming Buenos Aires Argentina; ^6^ Thoracic Oncology Program Memorial Cancer Institute/Florida International University Miami Florida USA; ^7^ Hematology and Oncology Department Hospital Militar Central Bogotá Colombia; ^8^ Oncology Department Instituto Nacional de Enfermedades Neoplásicas – IneN Lima Peru; ^9^ Medical Oncology Department Hospital San Juan de Dios San José Costa Rica; ^10^ Oncology Department Clínica Colsanitas Bogotá Colombia; ^11^ Thoracic Oncology Unit Hospital Italiano de Buenos Aires Buenos Aires Argentina; ^12^ Section of Oncology CEMIC Buenos Aires Buenos Aires Argentina; ^13^ Oncology Department Hospital Alemán Buenos Aires Argentina; ^14^ Oncology Department Hospital Privado de la Comunidad de Mar del Plata Mar del Plata Argentina; ^15^ Oncology Department Hospital Austral de Buenos Aires Buenos Aires Argentina; ^16^ Thoracic Oncology Unit, Marlene and Stewart Comprehensive Cancer Center University of Maryland Baltimore MD USA; ^17^ Oncology Department Institute of Oncology – ICAL, Fundación Santa Fe de Bogotá Bogotá Colombia; ^18^ Coyote Research Group, Pangaea Oncology, Laboratory of Molecular Biology Quiron‐Dexeus University Institute Barcelona Spain; ^19^ Institut d'Investigació en Ciències Germans Trias i Pujol Badalona Spain; ^20^ Institut Català d'Oncologia, Hospital Germans Trias i Pujol Badalona Spain

**Keywords:** Adult, immunotherapy, lung neoplasms, neoplasms/drug therapy, programmed cell death 1 receptor/antagonists & inhibitors

## Abstract

**Background:**

To compare survival outcomes of patients with advanced or metastatic non‐small cell lung cancer (NSCLC) who received immunotherapy as first‐, second‐ or beyond line, versus matched patients receiving standard chemotherapy with special characterization of hyperprogressors.

**Methods:**

A retrospective cohort study of 296 patients with unresectable/metastatic NSCLC treated with either, first‐, second‐, third‐ or fourth‐line of immunotherapy was conducted. A matched comparison with a historical cohort of first‐line chemotherapy and a random forest tree analysis to characterize hyperprogressors was conducted.

**Results:**

Median age was 64 years (range 34–90), 40.2% of patients were female. A total of 91.2% of patients had an Eastern Cooperative Oncology Group (ECOG) performance score ≤ 1. Immunotherapy as first‐line was given to 39 patients (13.7%), second‐line to 140 (48.8%), and as third‐line and beyond to 108 (37.6%). Median overall survival was 12.7 months (95% CI 9.67–14 months) and progression‐free survival (PFS) of 4.27 months (95% CI 3.97–5.0). Factors associated with increased survival included treatment with immunotherapy as first‐line (*P* < 0.001), type of response (*P* < 0.001) and PD‐L1 status (*P* = 0.0039). Compared with the historical cohort, immunotherapy proved to be superior in terms of OS (*P* = 0.05) but not PFS (*P* = 0.2). A total of 44 hyperprogressors were documented (19.8%, [95% CI 14.5–25.1%]). Leukocyte count over 5.300 cells/dL was present in both hyperprogressors and long‐term responders.

**Conclusions:**

Patients who receive immune‐checkpoint inhibitors as part of their treatment for NSCLC have better overall survival (OS) compared with matched patients treated with standard chemotherapy, regardless of the line of treatment.

## Introduction

Non‐small cell lung cancer (NSCLC) continues to be the leading cause of cancer‐related mortality in the world. With an estimated incidence of 2.09 million new cases and 1.76 million deaths in 2018 it leads the list for new cases and deaths among other neoplastic diseases. Survival at five years is estimated at 17.8%, which accounts for one of the greatest fatality rates in nontransmissible diseases.^1^, ^2^, ^3^, ^4^ In the era of precision medicine, patients with advanced/metastatic NSCLC are treated according to the presence or absence of molecular drivers including, but not limited to, *EGFR*, *BRAF* and *HER2* mutations or *ALK*, *NTRK1‐3* or *ROS‐1* rearrangements.^5^, ^6^, ^7^, ^8^

Nonetheless, a significant proportion of NSCLC patients present without targetable alterations. In such cases, immune checkpoint inhibitors (ICIs) have become the mainstay of treatment. Although initially proven effective in patients previously treated with platinum‐based chemotherapy, sufficient data from phase III trials (Keynote 024, 407 and 189) have standardized their use as first‐line treatment for NSCLC patients with negative drivers, either as single agents or in combination with chemotherapy, regardless of PD1 expression.^9^, ^10^, ^11^ More recently, data from CheckMate 227 has postulated tumor mutation burden as a promising selection tool for patients without molecular drivers between first‐line chemotherapy versus first‐line immunotherapy.^12^

Pembrolizumab, an anti PD‐1 IgG4 monoclonal antibody, showed efficacy in the phase II/III Keynote 010 study compared to docetaxel as a second‐line agent with better objective response rates, overall survival (OS) and toxicity profile than chemotherapy. The greatest response was observed in patients who harbored tumor PD‐L1 expression greater than 50% but was extended to those with expression >1%.^13^ Further studies, specifically the Keynote 024, demonstrated that pembrolizumab as a first‐line agent had greater efficacy compared with conventional chemotherapy in patients with PD‐L1 > 50%, similar to previous results.^14^

Nivolumab, another IgG4 monoclonal antibody, directed against PD‐1 has also been proven effective as a second‐line agent over docetaxel. Interestingly, nivolumab also demonstrated superiority in overall survival (OS), progression‐free surval (PFS), response rates (RR) and safety profile, for both squamous and nonsquamous (NS) histologies. In the case of NS, PD‐L1 negative patients appeared to lack therapeutic benefit.^15^, ^16^, ^17^ These results have not been translated into first‐line treatment.^18^

Atezolizumab, an IgG1 monoclonal antibody, with the same target as pembrolizumab, offered prolongation of OS in patients with >1% PD‐L1 expression with a benefit that was present regardless of histology compared to docetaxel.^19^, ^20^ As combinations of ICIs with other agents are gaining ground as initial treatments, survival of a larger number of patients with advanced disease has shown improvement.^9^, ^10^, ^12^, ^19^, ^20^, ^21^, ^22^ Additionally, there is few data available on the efficacy of immunotherapy for NSCLC in Hispanic patients. This is particularly the case in the large randomized studies which included Non‐Hispanic white or Asian individuals as the main treated populations.^23^, ^24^ Therefore, more data about NSCLC immunotherapy outcomes in Hispanics are needed. The aim of this study was to compare the survival of a heavily pretreated cohort of Hispanic patients with NSCLC with immunotherapy and a cohort of treatment naive patients that received chemotherapy.

## Methods

### Study design

A multicenter retrospective cohort study was conducted which included patients diagnosed between June 2013 and January 2018. Inclusion criteria were patients over 18 years old with advanced/nonresectable or metastatic NSCLC (proven histologically) who were treated with immunotherapy agents such as ipilimumab, nivolumab, pembrolizumab, durvalumab or avelumab as monotherapy, a combination of agents or combined with chemotherapy as first‐, second‐ or further line of therapy. Patients were assessed for OS and PFS. Additionally, initial clinical laboratories and clinical information was collected within the p‐Platform of the Latin American Consortium for the Investigation of Lung Cancer (CLICaP) and assessed by the authors with a de‐identified database. Serial evaluations were performed on each patient which accounted for assessment of treatment response according to the RECIST criteria and adverse effects to the Common Terminology Criteria for Adverse Events (CTCAE) Version 4.0.^25^ This study was approved by the research committee of the Clínica del Country: IT‐201009781.

### Statistical analysis

For descriptive purposes, continuous variables were summarized as arithmetic means, medians or interquartile ranges. Categorical variables were reported as proportions with 95% confidence intervals (95% CIs). In order to determine if the benefit of immunotherapy was present beyond the first‐line of treatment and irrespective of the moment of administration, a comparison between these patients and a published historical cohort of patients with similar disease characteristics and adenocarcinoma histology who received treatment with chemotherapy was performed (platinum/pemetrexed/bevacizumab followed by maintenance pemetrexed/bevacizumab).^26^ In order to make the cohorts comparable, a Mahalanobis matching discrepancy was done. As a first step a logistic regression to determine imbalances between the cohorts was conducted. Variables considered as confounders were used to match and reduce observations in both cohorts to achieve homogeneity. Mahalanobis matching was chosen over propensity score matching due to the latter having a tendency to lead to random pruning as suggested by King and Nielsen.^27^ A second logistic regression was conducted on the newly grouped cohort, in order to assess any disparities that could have arisen from matching and pruning. Survival analysis was based on the Kaplan‐Meier methodology. Differences in terms of OS and PFS were estimated using the log‐rank test or milestone analysis when applicable. After evaluation of hazard rates proportionality, Cox regression was used to evaluate survival determinants. As a target population, hyperprogressors, defined as patients who presented with disease progression eight weeks after initiation of treatment^28^ were categorized with regression analyses and a random forest tree (RFT) evaluation in order to find factors associated with this phenomenon. Results were represented in a scatter plot with representation for median PFS, hyperprogressor state and leukocyte count. Statistical analyses were performed using SPSS version 23.0 (SSPSS, Inc., Chicago, IL, US) and R version 3.5.1 (The R Foundation, Vienna, Austria).

## Results

### Patients and tumor characteristics

**Figure 1:**
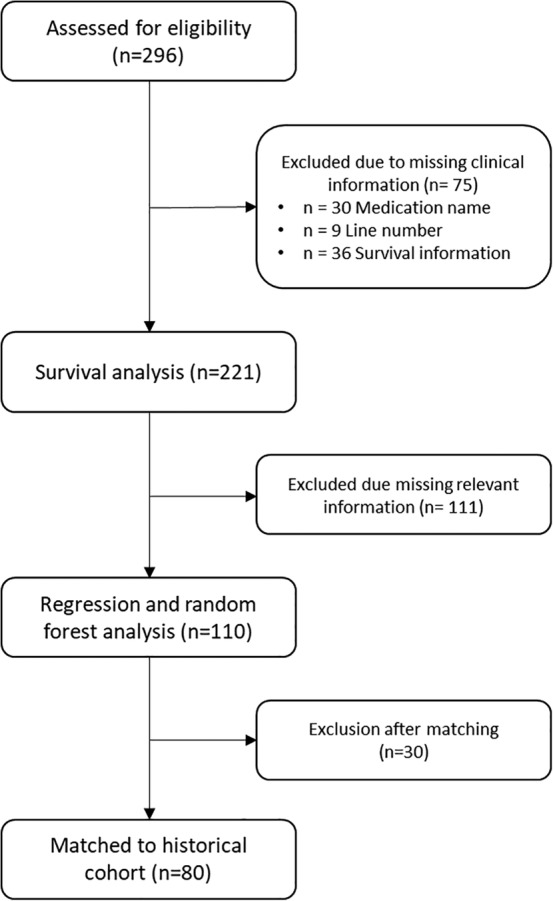
CONSORT flow diagram for study inclusion.

A total of 296 patients from reference centers in four countries (USA, Mexico, Colombia and Argentina) were included. Patients treated in the USA were included only if they were of Hispanic ethnicity. A summary of patients' characteristics is included in Table 1. Due to missing data several patients were excluded in each section of the study. For clarity a CONSORT flow diagram is presented in Figure 1.

**Table 1:** Patients' characteristics

Characteristic	Value	Range or 95% confidence interval
Median age	64 years	33–90
% of females	119 (40.2%)	95% CI (34.6%–45.8%)
ECOG	n (%)	95% CI
0	93 (31.5%)	25.3%–37.7%
1	177 (59.7%)	53.2%–66.2%
2	26 (8.8%)	5%–12.6%
Exposure to tobacco	n (%)	95% CI
Never/ever smokers	34 (23.4%)	19.1%–28.9%
Active/former smokers	94(75.6%)	70.8%–80.6%
Histology	n (%)	95% CI
Nonsquamous cell carcinoma	232 (90.6%)	87.1%–94.2%
Squamous cell carcinoma	28 (9.4%)	5.8%–12.9%
Metastatic involvement	n (%)	95% CI
Central nervous system	68 (23.1%)	17.5%–28.8%
Bone	136 (45.8%)	39.2%–52.5%
Liver	62 (20.8%)	15.4%–26.2%
Pleural	86 (28.9%)	21.1%–36.8%
Lymph nodes	226 (76.4%)	70.7%–82.5%
Suprarenal glands	62 (21.1%)	14%–28.1%
EGFR+	38 (12.8%)	9%–16.6%

The majority of patients were male (59.8%) with a median age of 64 years (range 34–90). Other characteristics are detailed in Table 1.

### Treatment characteristics

All patients received chemotherapy in some form during their treatment course. Standard treatment with platinum duplet was offered to all patients as first‐line. For further lines, treatment medications were administered under the treating oncologist's discretion based on local and international guidelines.

The most commonly used treatment was nivolumab (200 patients 75.2%), followed by pembrolizumab (36 patients, 13.7%), docetaxel/pembrolizumab (six patients, 2.4%), avelumab (seven patients, 2.8%), ipilimumab/nivolumab (eight patients, 3%), durvalumab (four patients, 1.5%), carboplatin/pemetrexed/nivolumab (two patients, 1%) and atezolizumab/entinostat (one patient, 0.4%). Additionally, immunotherapy was offered as first‐line treatment to 39 patients (13.7%), as second‐line to 140 (48.8%), third‐line to 78 (27.1%) and as fourth and additional lines to the remaining 30 patients (10.4%).

### Immunotherapy cohort survival outcomes

**Figure 2:**
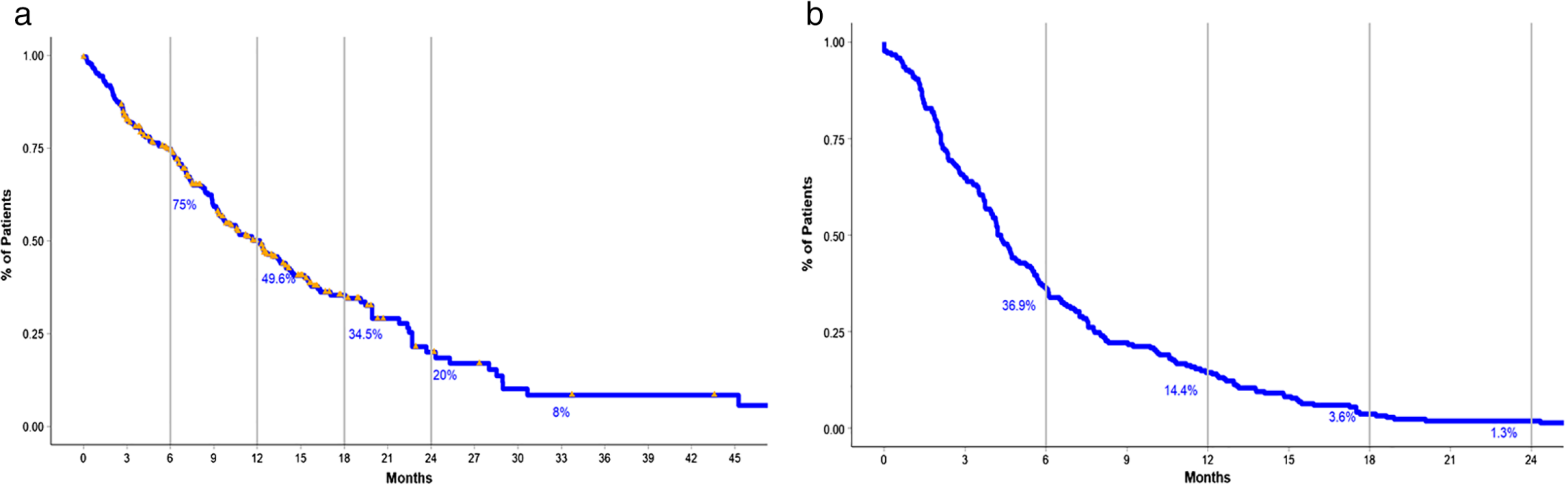
(a) Representation of overall survival after initiation of immunotherapy. Vertical lines represent milestone survival for six, 12, 18 and 24 months as a depiction of percentage of living patients. (b) Progression‐free survival with corresponding milestone survival after initiation of immunotherapy.

In terms of survival, median OS reached 12.17 months (95% CI 9.67–14 months) after initiation of immunotherapy. On the other hand, PFS was estimated around 4.27 months (95% CI 3.97–5.0) (Fig 2a,b). No differences between type of immunotherapy were found (*P* = 0.5). Treatment with ICIs as a first‐line treatment was not associated with a longer survival, with patients in this category reaching a median OS of 19.9 (95% CI 13.4–22.7) months and PFS of 4.86 (95% CI 3.06–17.25) months, compared to 12.2 months OS (95% CI.9.33–15.3) and 4.65 months PFS (95% CI 3.97–6.07) for second‐line and 9.2 months OS (95% CI 7.33–13.1) and 4.13 months PFS (95% CI 3.68–5.5) for third and further lines of treatment (*P* = 0.09 for OS and *P* = 0.1 for PFS). Additionally, immunotherapy as a first‐ or second‐line strategy was associated with greater overall response rates (45.7% and 29.5%, respectively, *P* = 0.002) compared to third and further lines. High PD‐L1 expression was present in 85.7% of the first group, followed by 63.4% and 41.7% in the second and latter (*P* = 0.005).

**Figure 3:**
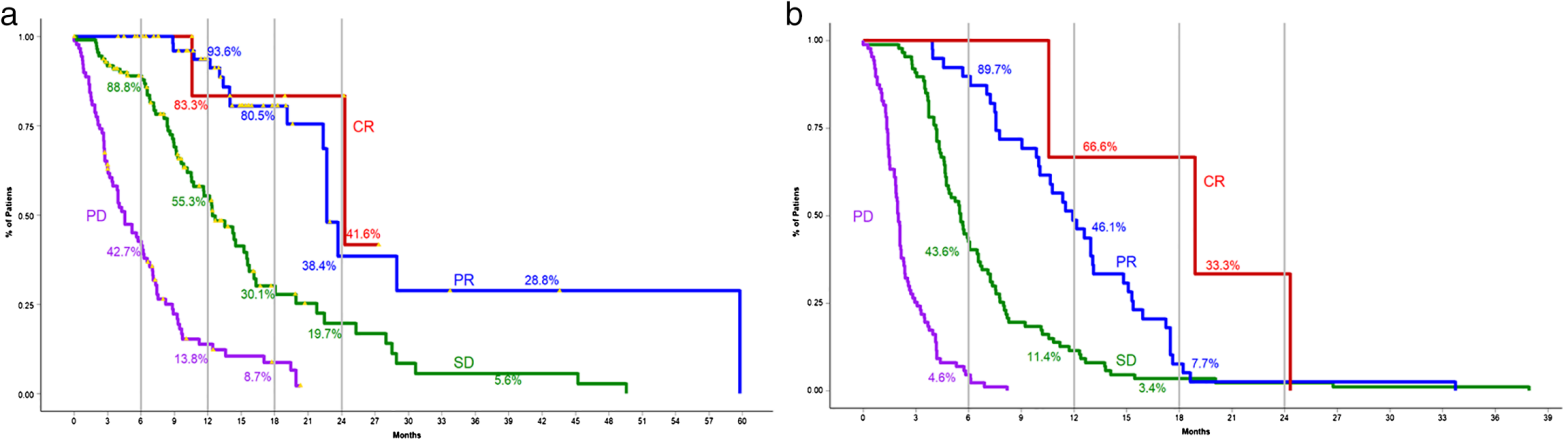
Survival according to response to immunotherapy. (a) Overall survival according to response for patients who were treated with immunotherapy who achieved complete response (CR, red line), partial response (PR, blue line), stable disease (SD, green line) and progressive disease (PD, purple line). Milestone survival is also represented by the vertical lines. (b) Progression‐free survival according to response for patients who were treated with immunotherapy who achieved complete response (CR, red line), partial response (PR, blue line), stable disease (SD, green line) and progressive disease (PD, purple line). Milestone survival is also represented by the vertical lines.

The strongest associated factor with both OS (HR 0.39 [95% CI 0.18–0.84]) and PFS (HR 0.07 [95% CI 0.02–0.19]) was type of response to immunotherapy (Fig 3 **)**. A more favorable and stronger response to ICIs was positively associated with male gender (*P* = 0.0397), absence of bone and pleural metastases (*P* = 0.031 and 0.165 respectively), high PD‐L1 status (*P* = 0.032) and immunotherapy as first‐line treatment (*P* = 0.0138). Metastatic sites were also associated with different outcomes in survival (Table 2 **)**. A forest plot representing factors associated with OS is presented in the Figure S1.

**Table 2:** Metastatic site and their association with survival

Site	HR	95% CI		*P*‐value
OS				
Bone	2.023	1.349	3.035	<0.001
PFS				
Liver	0.607	0.406	0.908	0.015
CNS	1.563	1.076	2.269	0.019
Nodal	0.626	0.433	0.903	0.012

### Matched cohort survival outcomes

**Figure 4:**
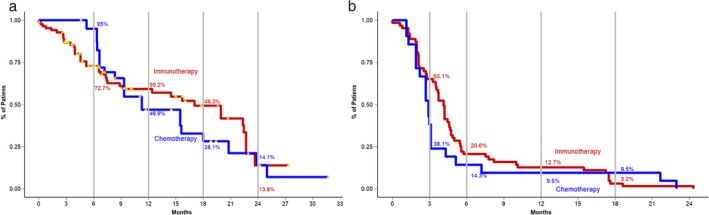
(a) Overall survival for patients treated with immunotherapy (red line) or chemotherapy (blue line) who were included in the matched cohort. (b) Progression‐free survival for patients treated with immunotherapy (red line) or chemotherapy (blue line) who were included in the matched cohort.

Logistic regression between the historical and the present cohorts revealed imbalances in the presence of hepatic (*P* = 0.016), pleural (*P* = 0.016), lung/suprarenal (*P* < 0.001) and absolute number of metastasis (*P* = 0.032). After matching, the pruning of a total of 30 observations in the immunotherapy treated and 104 in the historical cohort yielded a new database of 138 observations. Percent balance improvement of 100% was achieved for liver and pleural metastasis, whereas lung/suprarenal and absolute number of metastasis reached 86.46% and 70.47%, respectively. Second logistic regression on the new matched cohort revealed no imbalances. Survival analysis for unmatched data showed a clear advantage for administering platinum/pemetrexed/bevacizumab in terms of median OS (21.4 months [95% CI 18.0‐NR]) over immunotherapy (17.1 months [95% CI 9.2–22.7]); *P* = 0.002]). No benefit was observed in terms of PFS (4.2 months [95% CI 3.1–5.9] vs. 4.13 months [95% CI 3.52–4.67]); *P* = 0.2. After matching, a difference favoring the administration of immunotherapy was found. Median OS for immunotherapy remained identical, whereas for chemotherapy, after excluding patients with lower disease burden, not present in the immunotherapy cohort, resulted in a median OS of 11.3 months (95% CI 9.33–20.8), *P* = 0.05. PFS was also reduced in the historical cohort (2.9 months [95% CI 2.2–4.3]), not achieving a statistically significant difference (*P* = 0.3) (Fig 4 **)**.

### Hyperprogressors

Hyperprogressive disease was present in 44 patients (19.8%, [95% CI 14.5%–25.1%]) (Table 3 **)**. RFT analysis revealed a threshold of 5300 cells/dL in the leukocyte count was required for the presentation of hyperprogressive disease. Figure S2 represents the relationship between absolute leukocyte count at the beginning of immunotherapy and PFS. It is also worth noticing that long‐term responders begin to appear also around the 5300 cells/dL leukocyte mark. Spearman coefficient yielded a poor correlation (0.211) between absolute leukocyte count and PFS. On the other hand, the cumulative incidence of survivors over six months and white blood count were moderately correlated (0.649).

**Table 3:** Factors related to hyperprogression

Variable	OR	95% CI		*P*‐value
CNS metastasis	1.533	1.228	1.913	0.0009
Bone metastasis	1.515	1.172	1.959	0.004
Weight loss	1.53	1.176	1.991	0.004

### Toxicities

Distribution of adverse effects grade III and IV, evaluated at day 60 determined that fatigue was present in 26% of patients treated with immunotherapy in any line, followed by hypothyroidism which was the most common immune‐related side effect, occurring in 7.5% of patients. Pneumonitis affected 5.8% of patients, nephritis was present in 1.4% and hypophysitis in two cases.

## Discussion

When analyzing the survival curves of the present cohort and comparing the results to the previous publications, several key points are noted. First of all, the administration of immunotherapy as a first‐line treatment is crucial in achieving the greatest responses. When analyzing the results of the Keynote 024 trial beyond the fact that pembrolizumab was effective as a first‐line treatment, the allowance of crossover after progression between groups means this curve functionally represents a sequential treatment. In our cohort, patients who underwent immunotherapy as a first‐line treatment included 39 patients who archived a median OS of approximately 19.9 months, with 91% patients alive at six months. Close to results from KN‐024, patients who received immunotherapy after progression to chemotherapy achieved a median OS of 17 months, with 75% alive at six months. The increased survival at six months in this first setting is difficult to explain with the current data. Taking into account that high PD‐L1 expression (>50%) was around 86% in the first‐line group compared to 100% in the reference clinical trial, expression solely cannot explain the difference. Another possible explanation is that patients in this group also received combination therapy with either carboplatin/pemetrexed/pembrolizumab or ipilimumab/nivolumab. These treatments have proven to be highly effective and therefore could potentially increase survival.^9^, ^10^, ^12^, ^14^ Moreover, no differences between the stage at which the immunotherapy was administered (first‐line, second‐line, etc) and survival were found. A hypothesis regarding this aspect could be that other immunotherapy agents were administered after initial treatment failure, causing a delayed survival benefit in this subset of patients. Moreover, it is worth noting that treatment line was positively associated with response to treatment, suggesting that the expected benefit would be in this aspect. Furthermore, response to treatment was also influenced by metastatic site. Also, strengthening the observations published by Bensch *et al*.^29^ which revealed that the maximal tumoral uptake of ^89^Zr‐atezolizumab was observed by liver and nodal lesions, their occurrence also correlated with survival in this study.

Another interesting aspect in this work was the comparison among immunotherapy‐treated patients with a historical chemotherapy‐treated cohort. Although it would be difficult to assume a direct parallel even after statistical matching, this juxtaposition opens the door for a discussion. First of all, the historical cohort which consisted of patients with adenocarcinoma histology treated with a first‐line of solely platinum/pemetrexed/bevacizumab could be considered as immunotherapy naïve. On the other hand, the present cohort consisted in its majority of heavily pretreated patients. Despite the fact that follow‐up time was measured at the moment they started to receive immunotherapy, previously administered chemotherapy or a number of different therapies could limit the effectiveness of these medications. Even though this comparison seems unfair favoring the chemotherapy group, immunotherapy proved to be superior. This is particularly important in clinical practice where resources and medication availability are limited and could cause delays in administration of newer treatments. These results suggest that it is better to receive immunotherapy at some point during treatment in contrast to relying merely on chemotherapy and not receiving immunotherapy at all.

Another crucial aspect of immunotherapy consists of the unique negative phenomenon known as hyperprogressive disease. Hyperprogressive disease has a negative impact on survival in the initial treatment period. This phenomenon is responsible for the apparent detrimental effect compared with chemotherapy in the first few weeks as documented in both clinical trials and in the presented matched cohort.^30^ In the initial report of this phenomenon, the definition was set as a two‐fold increase in the tumor growth rate (TGR) after initiation of treatment, compared to pretreatment growth.^31^ This definition has evolved including a 50% increase in TGR.^32^ Nowadays, it is a three point definition that includes a time to treatment failure of two months, the aforementioned increase in TGR and a two‐fold or greater increase in progression growth.^33^ These variations in definitions cause heterogeneity in the studies evaluating this phenomenon and could potentially lead to different incidence rates depending on the definition used. Frequency of occurrence has been reported to be approximately 9%,^34^ with other groups describing 17%.^35^ In our cohort, the incidence rate was 19.8%. Our data is close to the upper limit, but considering the cohort and ethnical differences, this result could be influenced by these characteristics. Additionally, multiple studies have tried to determine clinical variables related to HPD in order to limit exposure to potential sufferers. The study published by Champiat *et al*. which included patients with different diagnoses found only a positive association in older patients.^34^ Positive associations were limited to MDM2/4 and EGFR amplifications in another cohort^33^ and no association in another.^35^ A possible hypothesis would be primary resistance to anti‐PD‐1/PD‐L1 therapy. The STK11 gene encodes for the serine/threonine kinase 11 which, when inactive, has been shown to limit the density of tumor infiltrating lymphocytes and therefore prevent an effective response to immunotherapy, especially in *KRAS*‐mutant tumors.^36^ Although now considered to be a strong and possible causal factor in ICIs' resistance, its role as a contributing factor in HPD is still unknown. Finally, based on our results and taking into account a leukocyte threshold for both the presence of longer responders and hyperprogressors, new hypotheses can be developed. First of all, a relatively higher number of leukocytes could correspond to a wider and diverse repertoire of both proinflammatory and anti‐inflammatory cell populations. On one hand, inflammation and modification of the tumor microenvironment could lead to immune compensatory mechanisms that eventually could result in malignant cell escape. It has been hypothesized that NFATC1 and NF‐kB p65 activity increase after PD‐L1 inhibition, both being pro oncogenic modulators. Additionally, secondary compensatory activation of the Ras‐MAPK and PI3K/AKT pathways could play a synergic effect.^37^, ^38^ On the other hand, it has also been demonstrated that the presence of senescent T CD4 (Tsens) lymphocytes (CD27 and CD 28 negative) is a strong marker for response. Interestingly, patients with populations of Tsens higher than 40% experience the greatest benefit, whereas patients with lower counts are more likely to experience disease progression during treatment. Additionally, PD‐1/L1 blockade correlates with changes in circulating Tsens count. The ideal course of treatment would be a decrease in number, with the majority of responders experiencing this change. Moreover, an increase in the number of Tsens is observed in all patients suffering from HPD.^39^ All in all, these considerations suggest that response to immunotherapy can either follow two possible pathways; one leading to the inactivation of Tsens and causing a cytotoxic response, or the other to overactivation which leads to the proliferation of Tsens and additionally, triggering a molecular compensatory response to PD‐L1 blockade and promoting tumor growth. Our findings of the leukocyte count threshold seem to indicate that a quantitatively functioning immune system is a prerequisite for both events. Interestingly, the lack of patients who experienced HPD or PFS beyond six months with leukocyte counts <5000 cells/dL could potentially be explained by this hypothesis. Additional contributions of this study, as mentioned previously, consist of the publication of the largest cohort of Hispanic NSCLC patients treated with immunotherapy, validating the benefits in terms of survival, at least compared with historical cohorts. This is important due to the lack of literature for this population.

This study carries some limitations which should be considered when interpreting the data. Due to its retrospective nature and the heterogeneity of treatments that patients received, the results differ slightly from published randomized controlled trials. Additionally, missing data, including a detailed characterization of previously administered treatments, challenges the conclusions.

In conclusion, immunotherapy for the treatment of patients with advanced/metastatic NCSLC is a definitive alternative to chemotherapy and has benefits that become apparent at any moment of the disease course, although their magnitude becomes smaller as treatment administration is delayed.

## Disclosure

The authors report no conflict of interest.

## Supporting information


**Figure S1.** Subgroup analysis of factors associated with overall survival (OS). For comparison, Hazard ratios calculation was conducted against the initial category in each subgroup. HR, Hazard ratio; LBCI, Lower bound of 95% confidence interval; UBCI, upper bound of 95% confidence interval.

**Figure S2.** Relationship between leukocyte count and response type.
