# Supplementary figures and images for: Immunotherapy at any line of treatment improves survival in patients with advanced metastatic non‐small cell lung cancer (NSCLC) compared with chemotherapy (Quijote‐CLICaP)
Source: Thorac Cancer. 2019 Dec 12;11(2):353–61. doi: 10.1111/1759-7714.13272 (PMC6996989; doi:10.1111/1759-7714.13272)

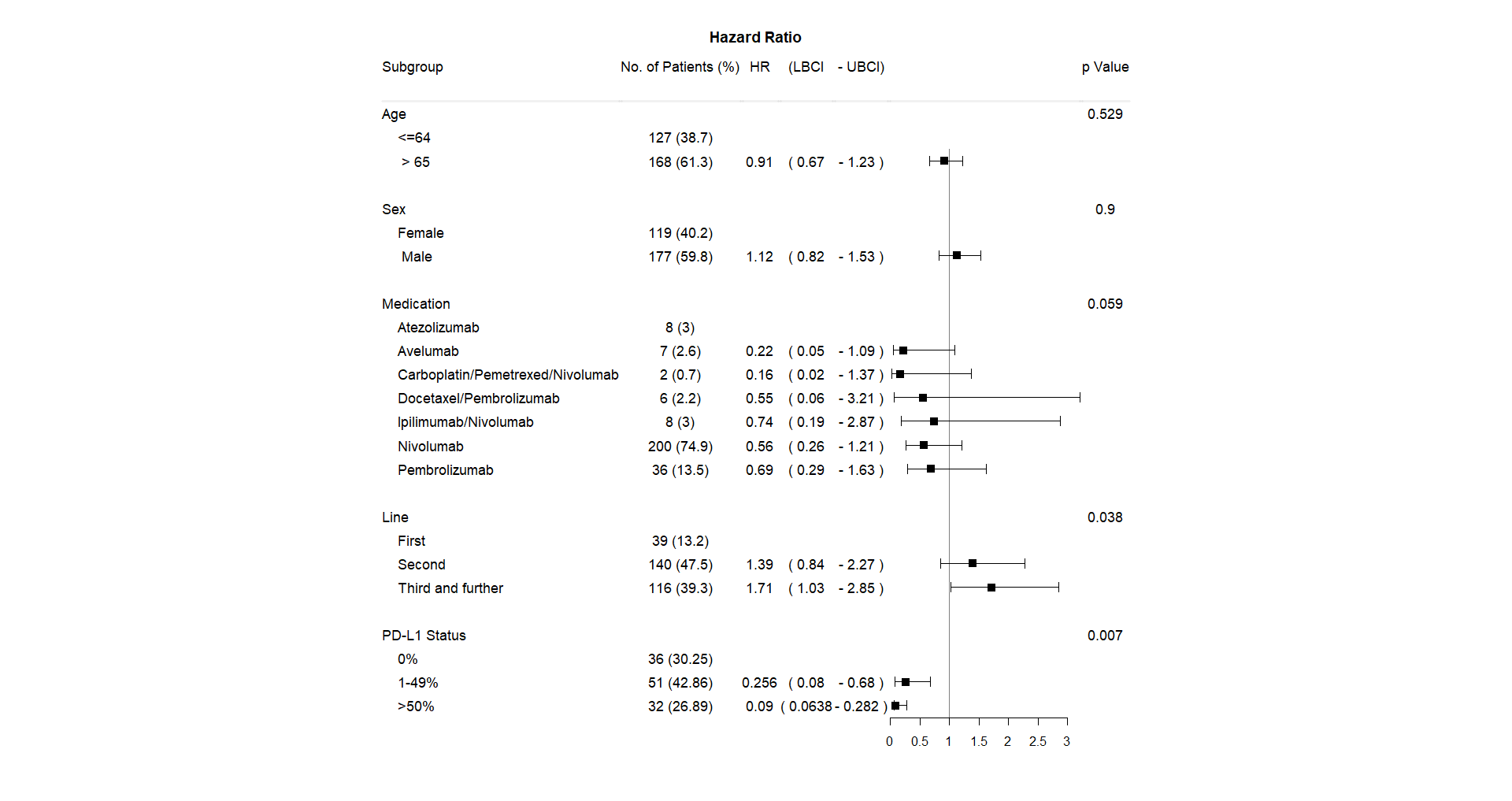

Supplement: Supplementary file 1 — Figure S1. Subgroup analysis of factors associated with overall survival (OS). For comparison, Hazard ratios calculation was conducted against the initial category in each subgroup. HR, Hazard ratio; LBCI, Lower bound of 95% confidence interval; UBCI, upper bound of 95% confidence interval. [file TCA-11-353-s001.png]

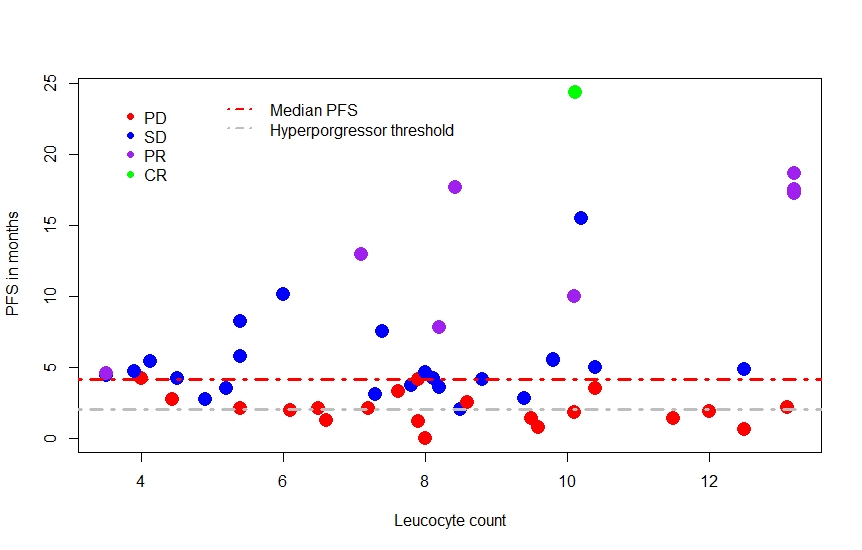

Supplement: Supplementary file 2 — Figure S2. Relationship between leukocyte count and response type. [file TCA-11-353-s002.jpg]
